# Novel methods of testing and calibration of oscillometric blood pressure monitors

**DOI:** 10.1371/journal.pone.0201123

**Published:** 2018-08-06

**Authors:** Branko G. Celler, Ahmadreza Argha, Phu Ngoc Le, Eliathamby Ambikairajah

**Affiliations:** Biomedical Systems Research Laboratory, School of Electrical Engineering and Telecommunications, University of NSW, Sydney, NSW, Australia; University of Perugia, ITALY

## Abstract

We present a robust method for testing and calibrating the performance of oscillometric non-invasive blood pressure (NIBP) monitors, using an industry standard NIBP simulator to determine the characteristic ratios used, and to explore differences between different devices. Assuming that classical auscultatory sphygmomanometry provides the best approximation to intra-arterial pressure, the results obtained from oscillometric measurements for a range of characteristic ratios are compared against a modified auscultatory method to determine an optimum characteristic ratio, Rs for systolic blood pressure (SBP), which was found to be 0.565. We demonstrate that whilst three Chinese manufactured NIBP monitors we tested used the conventional maximum amplitude algorithm (MAA) with characteristic ratios Rs = 0.4624±0.0303 (Mean±SD) and Rd = 0.6275±0.0222, another three devices manufactured in Germany and Japan either do not implement this standard protocol or used different characteristic ratios. Using a reference database of 304 records from 102 patients, containing both the Korotkoff sounds and the oscillometric waveforms, we showed that none of the devices tested used the optimal value of 0.565 for the characteristic ratio Rs, and as a result, three of the devices tested would underestimate systolic pressure by an average of 4.8mmHg, and three would overestimate the systolic pressure by an average of 6.2 mmHg.

## Introduction

The gold reference standard for blood pressure (BP) measurement is based on direct measurement of BP via an intra-arterial catheter placed in the radial artery [1,2]. However, because this invasive procedure is inconvenient and involves some risk to the patient, an alternative compromise solution is based on classical sphygmomanometry where a cuff placed on the upper arm is inflated to a pressure well above systolic pressure and then allowed to deflate at a steady 2-3mm per second. A stethoscope is placed over the brachial artery just below the edge of the occluding cuff and as the cuff pressure falls, blood begins to flow and Korotkoff sounds are heard. The first Korotkoff sound defines the systolic pressure point, and the disappearance of the Korotkoff sounds, as the cuff is further deflated, defines diastolic pressure.

Numerous studies [3–8] point out that there are considerable inter-operator differences in estimating blood pressure using classical sphygmomanometry, particularly with the determination of diastolic pressure [5,6]. In previous studies [3,7], we presented data to suggest that the accuracy of sphgmomanometry is dependent on (i) the hearing acuity of the operator, (ii) the amplitude and particular waveform morphometry of the Korotkoff sounds, and (iii) the sensitivity of the stethoscope.

Despite these inherent limitations, the Association for the Advancement of Medical Instrumentation (AAMI) [9] and the British Hypertension Society (BHS) [10] recommend sphygmomanometry as the gold reference for the calibration of non-invasive blood pressure (NIBP) monitors, with two trained operators simultaneously recording and then averaging their individual estimates of systolic and diastolic pressures.

An NIBP measuring device would comply with the American National Standards Institute (ANSI)/AAMI SP-10 standard [9] if its measurement error has a mean error of no more than 5 mmHg, and a standard deviation of no more than 8 mmHg. The BHS protocol [10] also provides a classification of the NIBP measuring devices based on their accuracy. In particular, a grade A device must have at least 60% of the measures within an error of 5 mmHg, 85% of the measures within an error of 10 mmHg, and 95% within 15 mmHg. We note that these standards actually permit a substantial margin for error as a standard deviation of 8 mmHg means that 32% of data can be in error by more than 8 mmHg.

### The oscillometric method

The majority of NIBP monitors available on the market today are based on the oscillometric method [11–18], because of its simplicity and robustness. The oscillometric method requires the inflation of the cuff beyond the anticipated systolic pressure. As the cuff deflates, the oscillometric waves superimposed on the pressure measurement are processed to produce the oscillometric waveform envelope (OMWE). In the conventional maximum amplitude algorithm (MAA), the maximum value of the OMWE is thought to correspond closely with the mean arterial pressure (MAP) [12]. The systolic point is then found at about 50% of the peak height (Rs = 0.5) on the rising phase of the envelope and the diastolic point is found at about 70% of the peak height (Rd = 0.7) on the falling phase of the envelope [15]. However, the best values for these characteristic ratios are disputed, with values for Rs and Rd reported in the range of [0.45 0.73] and [0.69 0.83], respectively [16]. Moreover, these empirical ratios are sensitive to changes in physiological conditions, including most importantly the pulse pressure (systolic minus diastolic blood pressure), the degree of arterial stiffness and the pulse rate [5,12,14–18].

Specific algorithms or characteristic ratios used in commercially available devices however are closely guarded trade secrets that are not subject to independent critique and validation. As every NIBP monitor may well implement different algorithms for the determination of systolic and diastolic pressure, commercially available simulators make it clear that it is unrealistic to expect values that are identical, or even very close, to their own pre-set values on the simulator, and indeed they do not report on what algorithms or characteristic ratios are used internally. If the pre-set values of systolic and diastolic pressure set on the simulator are identical to those recorded in the device under test, we would indeed expect that the algorithms and characteristic ratios used in the NIBP monitor under test would be identical to those used in the simulator.

Curve fitting of the oscillometric waveform envelope is a vital first step for all MAA in BP measurement. Popular methods in recent years include linear or polynomial models [19], generalised curve fitting [20], or the fitting of asymmetric Gaussian or Lorentzian functions [21]. Curve fitting can also be supplemented using artificial neural network [22] or fuzzy logic [23] to minimise the MSE.

### Comparison of NIBP oscillometric method against intra-arterial measurement

Comparisons of NIBP measurements, against intra-arterial measurements, generally give poor results [24,25]. In a large study [24] of 986 patients and 26,942 records, where BP was recorded simultaneously via a brachial artery catheter, and noninvasively using the oscillometric method with a brachial sphygmomanometer cuff, mean values of diastolic and systolic pressures were in good agreement, but correlation coefficients (r) between invasive systolic blood pressure (SBP) and non-invasive systolic blood pressure, and between invasive and non-invasive diastolic blood pressure (DBP) measurements were 0.6 and 0.45, respectively. The average differences of systolic and diastolic blood pressure were 2.8 ± 27.2 mmHg and 8.9 ± 20.9 mmHg between invasive BP (IBP) and NIBP. Ignoring the mean errors, the large standard deviations indicate that 32% of systolic and diastolic NIBP measurements were in error by more than ±27.2 mmHg and ±20.9 mmHg, respectively.

## Aims

In Phase 1 of this study we use an industry standard NIBP simulator to determine the characteristic ratios used in a number of NIBP monitors available in the market and to explore differences between them. In Phase 2, we then make the assumption that classical auscultatory sphygmomanometry provides the best approximation to intra-arterial pressure [1,2,4,5,8,9], and directly compare the results obtained from oscillometric measurements for a range of characteristic ratios, against a modified auscultatory method [3,7]. This method accurately determines systolic pressure from simultaneous recordings of the blood pressure trace, the oscillometric waveforms and the Korotkoff sounds. We then identify the best characteristic ratio that provides estimates of systolic pressure using the oscillometric method closest to the reference values for systolic pressure obtained from the modified auscultatory method.

## Materials and methods

Data used in this paper has been deposited with Research Data Australia (https://researchdata.ands.org.au/) and can be accessed using the following Identifier: (http://handle.unsw.edu.au/1959.4/resource/collection/resdatac_670/1)

### Phase 1. Determination of characteristic ratios using an industry standard simulator

**Fig 1:**
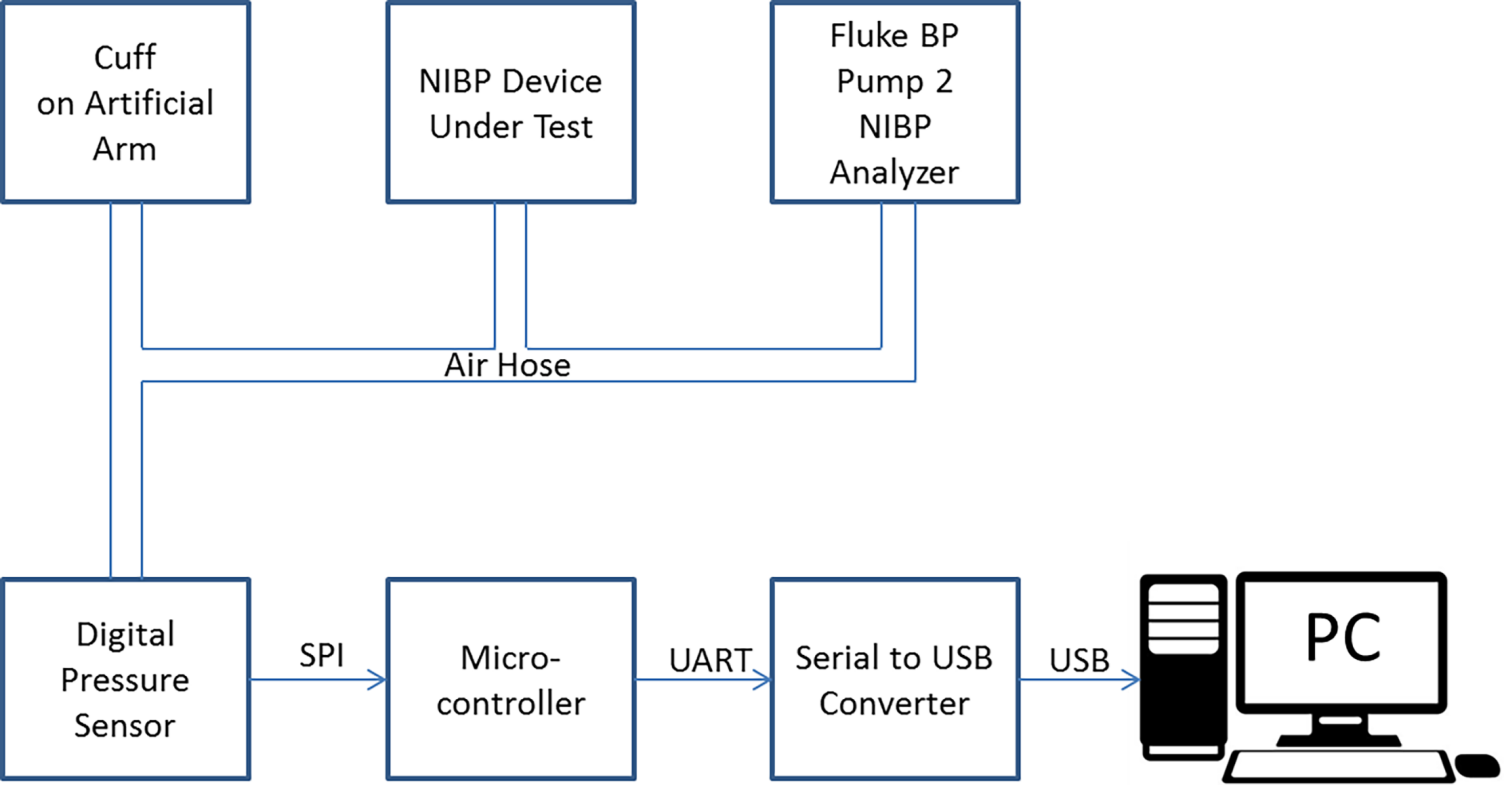
Schematic diagram of testing configuration for NIBP oscillometric monitors using the Fluke BP NIBP Analyser.

We use a Fluke BP Pump2 NIBP Analyser, to test the calibration and then derive the characteristic ratios for a number of commercially available NIBP monitors. This analyser provides repeatable dynamic blood pressure simulations, static calibration, automated leak testing, and pressure relief valve testing and allows the operator to verify the performance claims of different blood pressure monitors. A stepper motor and lead screw moves a piston into the manifold to decrease the manifold volume, thereby creating pressure pulses of 0.5–1.5cc to simulate a human subject. The cuff of the NIBP monitor under test is wrapped around a compliant mandrel to simulate an artificial arm. The tester generates completely repeatable simulations. Our experimental setup is shown schematically below in Fig 1.

The air hose of the NIBP monitor under test was connected via T connectors to the Fluke BP Pump 2 NIBP Analyser and an artificial arm as well as an independently calibrated digital pressure sensor, (SensorTechnics HCEM500GUH9P3) with 375 mmHg Full Scale. The output of the digital pressure senor was read via a microcontroller and a serial to USB converter into a personal computer. The output of the digital pressure sensor was carefully calibrated against the pressure provided by the Fluke Analyser and the calibration curve recorded for future use.

For each NIBP monitor under test, we selected three nominal systolic /diastolic test pressures at 90/50, 120/80 and 180/120 mmHg, which were programmed sequentially into the Fluke BP Pump 2 NIBP Analyser for each device.

### Data collection procedure

The target pressures, heart rate (HR) and pulse amplitude were configured on the Fluke Analyser, keeping HR at 80 bpm and oscillometric pulse volumes constant at 1.0 cc. The pumping and recording cycle was started by pressing the start button on the NIBP monitor. The Fluke Analyser generates pulses when the cuff pressure is within the target systolic and diastolic pressure range. The data collecting process is as follows:

1. Set the target systolic and diastolic pressures, target HR and pulse amplitude (1.0 cc) on the Fluke Analyser.
2. Start data logging on the PC.
3. Press the start button on the NIBP monitor.
4. The NIBP monitor will pump up the cuff pressure higher than the selected systolic pressure, and will then deflate the cuff gradually.
5. The Fluke Analyser will add pulses when the cuff pressure is within target systolic and diastolic pressure range.
6. The NIBP monitor deflates the cuff completely when the cuff pressure is below the detected diastolic pressure.
7. Observe systolic, diastolic pressure and HR recorded on the NIBP monitor under test.
8. Stop data logging on the PC. Save logging data to a file.

**Fig 2:**
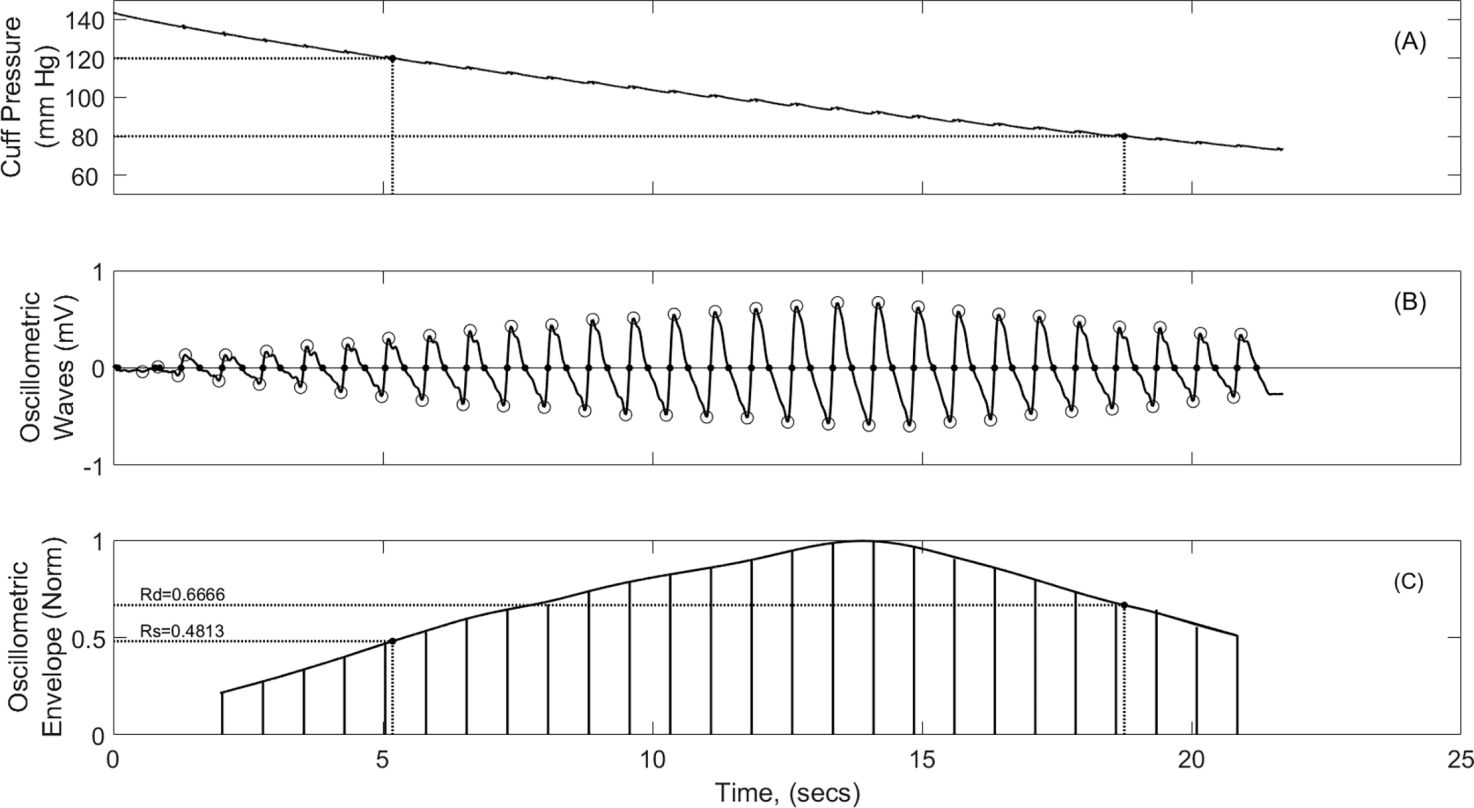
Development of Oscillometric Waveform Envelope (OMWE) and determination of characteristic points Rs, and Rd with SBP and DBP points set at 120/80 mm Hg.

Using the protocol outlined above we sampled the resulting oscillometric waves throughout the rising phase and the release phase of the pumping cycle at 500 samples/sec. We subsequently ignored the pumping up phase and applied a 0.7Hz finite impulse response (FIR) high pass filter followed by a 7Hz low pass filter to the remaining signal to eliminate noise from the internal stepper motor. Both filters were applied first in the forward direction and then in the reverse direction to avoid any phase distortion. We then used a sophisticated algorithm to accurately find the foot and the peak of each oscillometric wave and calculated the distance between the foot and the peak to produce the OMWE, as shown in Fig 2, panels B and C.

The OMWE was then normalised to its peak value and a cubic smoothing spline function with a smoothing parameter of 0.1 was used to fit a smooth curve (Panel C) to the oscillometric envelope. Characteristic ratios could then be read directly from the OMWE at the set systolic and diastolic pressures. If both the calibrator and the NIBP monitor under test used the same characteristic ratios, then the systolic and diastolic pressures dialled into the calibrator would be expected to be identical with the results reported on the NIBP monitor within a very small margin of error. However, if the results displayed on the NIBP monitor are significantly different from those dialled up on the Fluke Analyser, then the characteristic ratios and /or the algorithms used in the NIBP test device must be different from those built into the Analyser.

We analysed three commercially available Original Equipment Manufacturer (OEM) oscillometric NIBP monitors manufactured in China, one manufactured in Germany and two NIBP monitors manufactured in Japan. We tested each NIBP monitor at calibrator settings of 90/50, 120/80 and 180/120 mmHg, and produced the OMWE for each as shown in Fig 2, using both cubic spline and linear curve fitting procedures. For each test, we noted the BP measurement shown on the NIBP monitor, and from the OMWE we recorded the characteristic ratios Rs and Rd for the displayed systolic and diastolic pressures.

### Phase 2. Determination of optimal Rs against a reference database of blood pressure recordings

In two previous studies [3,7], we described the development of a database of 310 blood pressure recordings from 102 patients (71 males and 31 females) aged 50.7±21.5 years (range 21–96 years). (University of New South Wales Human Research Ethics Committee (HREC) Approval Number: 12/11). These recordings were selected from a larger database of 730 recordings to have no signal artefact or evidence of cardiac arrhythmia and to satisfy the distribution of age, gender and arm diameter recommended by the ANSI/AAMI SP10:2009 standard for electronic or automated sphygmomanometers. A further review of this database identified an additional six records where signal artefacts or arrhythmia were observed and these were rejected, leaving a reference database of 304 records.

**Fig 3:**
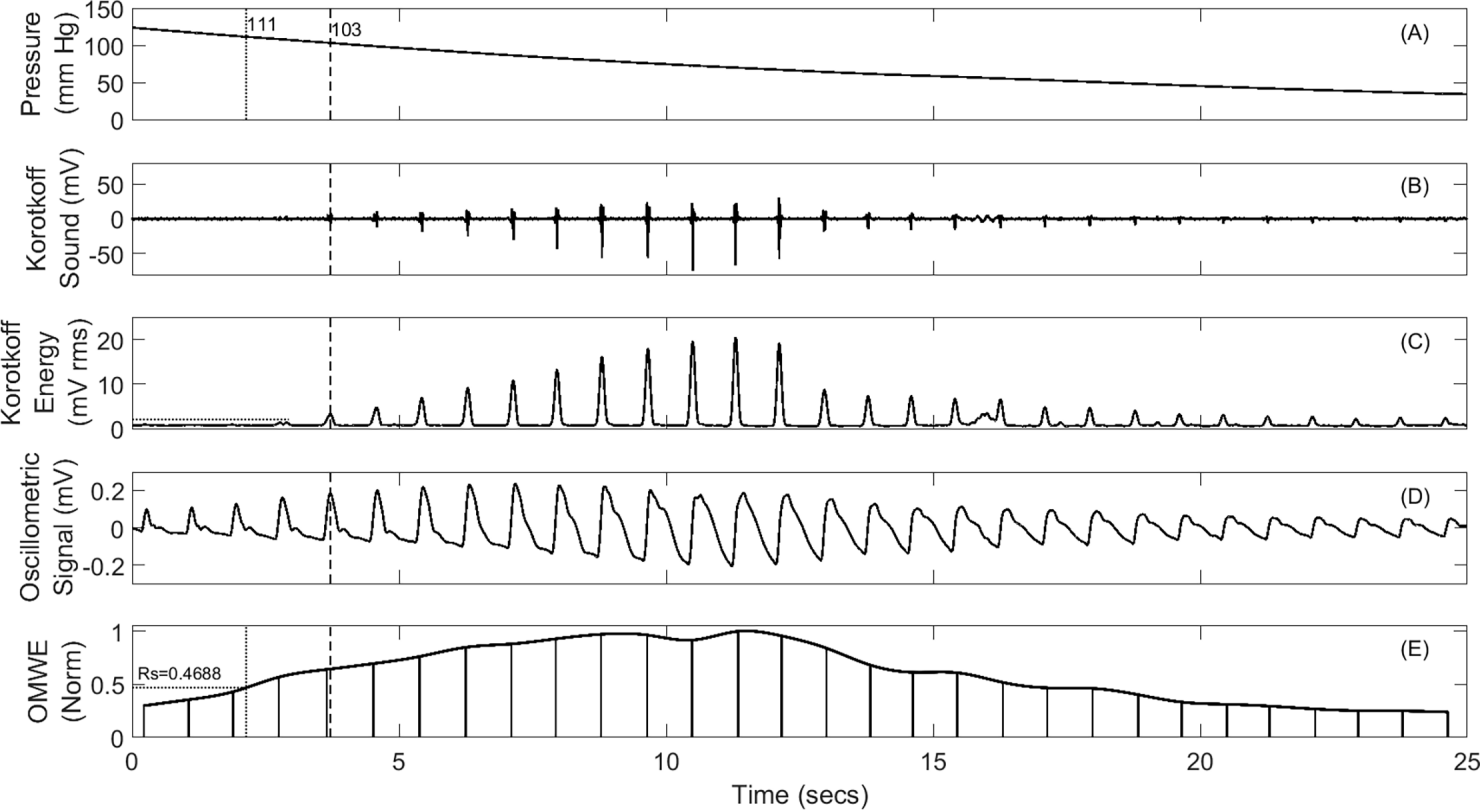
Sample blood pressure record (Panels A-D) from reference database and derived OMWE (Panel E). The vertical dashed lines points to the SBP at which the first Korotkoff sound appears.

The NIBP recording system used to develop the reference database was a commercially available multi-parameter clinical monitoring unit (CMU) from Telemedcare Pty Ltd (www.telemedcare.com.au). The NIBP module was used in its normal automated configuration which automatically inflates the BP cuff to a pre-set pressure and uses servo control to reduce the cuff pressure at a rate of 2–3 mm per second. A typical recording is shown in Fig 3.

As described in [3,7], for all 304 records, the mean noise level (MNL) with the cuff fully inflated was measured and a threshold value of MNL + 4×Standard Deviation (SD), shown in Fig 3 as a dotted horizontal line, was calculated. The cuff pressure at which the first Korotkoff sound exceeded this threshold was selected as the reference systolic pressure. All records were also checked visually for accuracy. The systolic pressure determined using this semi-automated method provides the reference database for comparison with systolic pressure estimates based on oscillometric methods.

As shown in Fig 3, for any of the 304 records available in the reference database, the OMWE can be formed and the oscillometric estimates of systolic pressure measured for any value of the characteristic ratio Rs. This then permits statistical analysis of the differences between the systolic pressure derived for any value of Rs against the reference value obtained using the semi-automated auscultatory method described above.

Although a simple algorithm was also developed to determine diastolic pressure, there is insufficient agreement [3,6] on the automated determination of diastolic pressure from these recordings to warrant considering diastolic pressure in this paper.

### Statistical methods

MATLAB (R2015B) was used for all data analysis. All data collected was tested for normality using the single sample Kolmogorov-Smirnov goodness of fit hypothesis test and visually verified using an empirical quantile-quantile plot of the sample quantiles of X versus theoretical quantiles from a normal distribution. If the distribution of X is normal, the plot will be close to linear. Categorical variables such as % changes were tested with a one sample t-test with a null hypothesis of “mean is zero”. When data samples were measured simultaneously the differences were tested for normality and a paired sample t-test applied with the null hypothesis that the two matched samples come from distributions with equal means. When data samples were not matched the two-sample t-test was used with the assumption that the data was normally distributed with unknown but equal variances. For data that was not normally distributed the Wilcoxon rank sum test for equal medians was used. We used Bland Altman plots to evaluate the bias between the mean differences between the oscillometric MAP method for determining systolic pressure and the reference value obtained using the semi-automated auscultatory method described earlier, to estimate the agreement interval between the two different methods, after testing that the differences were normally distributed.

## Results

Calibration of the digital pressure transducer was carried out against a range of pressures (75–300 mmHg) dialled up on the Fluke BP Pump2 NIBP Analyser. The resultant calibration curve is characterised by an intercept of 2740 digital counts and slope of 0.01722 (mmHg/Digital Count) with a P < 0.0001 and adjusted r^2^ = 0.9999878.

### Phase 1: Determination of characteristic ratios Rs and Rd from Fluke Analyser

The formation of the Oscillometric Waveform Envelope (OMWE) and determination of characteristic points Rs and Rd, as shown in Fig 2, were repeated for three different systolic and diastolic pressure combinations for different commercially available NIBP monitors as described in the methods. For purposes of comparison linear interpolation as well as cubic spline was also used to estimate the OMWE. For brevity we do not display the data for linear interpolation. In Table 1, we evaluate the characteristic ratios for six NIBP monitors available in the market.

**Table 1:** Determination of characteristic ratios Rs and Rd from Fluke Analyser for six NIBP devices using cubic splines to form the OMWE.

	Fluke Analyser Output				NIBP Device Output			
Dev	SBP (mmHg)	DBP (mmHg)	Rs	Rd	SBP (mmHg)	DBP (mmHg)	Rs	Rd
1	90	50	0.4841	0.6298	92	50	0.4386	0.6298
1	120	80	0.4792	0.6409	122	80	0.4402	0.6409
1	180	120	0.4792	0.6342	183	122	0.4292	0.6614
2	90	50	0.5006	0.6185	91	52	0.4835	0.6843
2	120	80	0.4926	0.6022	121	82	0.4726	0.7265
2	180	120	0.4816	0.6699	176	124	0.5243	0.7032
3	90	50	0.4772	0.5936	90	49	0.4772	0.6027
3	120	80	0.5071	0.6249	122	80	0.4393	0.6249
3	180	120	0.4823	0.6337	181	120	0.4566	0.6337
			0.4871^α^ (0.0106)	0.6275^β^ (0.0222)			0.4624^α^ (0.0303)	0.6564^β^ (0.0407)
4	90	50	0.4913	0.6827	81	50	0.6585	0.6827
4	120	80	0.4629	0.6332	107	81	0.7056	0.6567
4	180	120	0.4624	0.6531	166	123	0.6465	0.7030
5	90	50	0.5049	0.6432	82	54	0.6542	0.7707
5	120	80	0.4838	0.6340	110	85	0.6416	0.7814
5	180	120	0.4697	0.6150	164	130	0.7027	0.8065
6	90	50	0.5386	0.8511	86	49	0.6221	0.8192
6	120	80	0.4882	0.7408	116	79	0.5839	0.7188
6	180	120	0.4883	0.7375	171	124	0.6268	0.7922
			0.4874^χ^ (0.0178)	0.6878^δ^ (0.0761)			0.6491^χ^ (0.0383)	0.7479^δ^ (0.0587)

P^α^ = 0.0511

P^β^ = 0.0741

P^χ^<0.0001

P^δ^ = 0.0516

Characteristic ratios Rs and Rd were almost identical for all six NIBP devices irrespective of whether cubic splines or linear fitting was used to form the OMWE. Data using linear interpolation are not shown in Table 1 for brevity. Rs using cubic splines (0.5557±0.1017), was not significantly different (Paired t-test, P = 0.8653) from values derived using linear fitting (0.5554±0.1013). Similarly, there were no significant differences (Paired t-test, P = 0.8272) between values of Rd, derived using cubic splines (0.7021±0.0680) and those derived from linear fitting (0.7010±0.0665). We subsequently used cubic spline fits throughout the paper.

### Differences in systolic and diastolic pressures recorded

In Table 1, systolic and diastolic pressures measured by the Fluke analyser were in excellent agreement with the results recorded from the first three NIBP devices, with errors of -0.83±1.33% for systolic pressure and -1.06±1.94% for diastolic pressure, both well within the tolerances allowed by ISO 81060–2:2013(E), the AAMI equivalent [9] or the BHS standard [10]. In contrast, the equivalent errors for the last three NIBP devices were significantly larger, 7.5±2.62% for systolic pressure and -2.94±3.86% for diastolic pressure. These differences were significant (P<0.0001) for systolic pressure, but not statistically significant (P = 0.2106) for diastolic pressure.

### Characteristic ratios derived from Fluke BP Pump2 NIBP Analyser

Characteristic ratios derived from the NIBP Analyser did not vary significantly for different combinations of systolic and diastolic pressures for most of the six devices tested. The characteristic ratio Rs was almost identical across all six devices and eighteen measurements with a mean value of 0.4874±0.018, when a cubic spline was used to fit the OMWE.

It was noted however that for NIBP monitor 6, the mean value recorded for Rd, 0.7765±0.0647, was higher, but not significantly higher (P = 0.0637) than that recorded for the other five NIBP monitors tested (0.6339±0.0232), although no such difference (P = 0.3435) was observed for Rs. We also noted that device 6, unlike all other devices tested, had a different proprietary design for the cuff, based on a semi-rigid outer shell which made the cuff easier to place on the arm. This design feature did not influence the Rs ratio but appeared to influence the Rd ratio.

### Derivation of characteristic ratios based on device readings

The characteristic ratios calculated for the device reading of systolic and diastolic pressures were significantly different between the three Chinese manufactured devices and the remaining three. Rs for the first three devices was 0.4624±0.0303, significantly smaller (P<0.0001) than the Rs of 0.6491±0.0383 recorded for the last three devices tested. Similarly, Rd for the Chinese manufactured devices was 0.6564±0.0407, significantly smaller (P = 0.0018) than 0.7479±0.0587 calculated for the last three devices tested.

### Comparing Analyser and device readings

The characteristic ratio Rs for the Chinese manufactured devices obtained from the Fluke Analyser was 0.4871±0.0106, not significantly different (P = 0.0511) from the value of 0.4624±0.0303 obtained from the device output. For Rd the Analyser output was 0.6275±0.0222, not significantly different (P = 0.0741) from the values of 0.6564±0.0407 from the device output.

For the remaining three devices, the Characteristic ratio Rs derived from the Fluke Analyser was 0.4878±0.0237, significantly smaller (P<0.0001) than the value of 0.6491±0.0383 obtained from the device output. For Rd the Analyser output was 0.6878±0.0761, smaller but not significantly different (P = 0.0516) than the values of 0.7479±0.0587 from the device output.

### Phase 2: Determination of optimal characteristic ratio Rs against a reference database of patient blood pressure recordings

The systolic pressure for each of the 304 NIBP records in the reference database was calculated using a range of values of Rs, ranging from 0.45 to 0.73 as reported in the literature. This range included values of Rs = 0.4624, for the Chinese manufactured devices and Rs = 0.6491 for the other German and Japanese manufactured devices. For each Rs, all 304 files were processed and the differences tested for normality, before generating the Bland- Altman plots.

**Fig 4:**
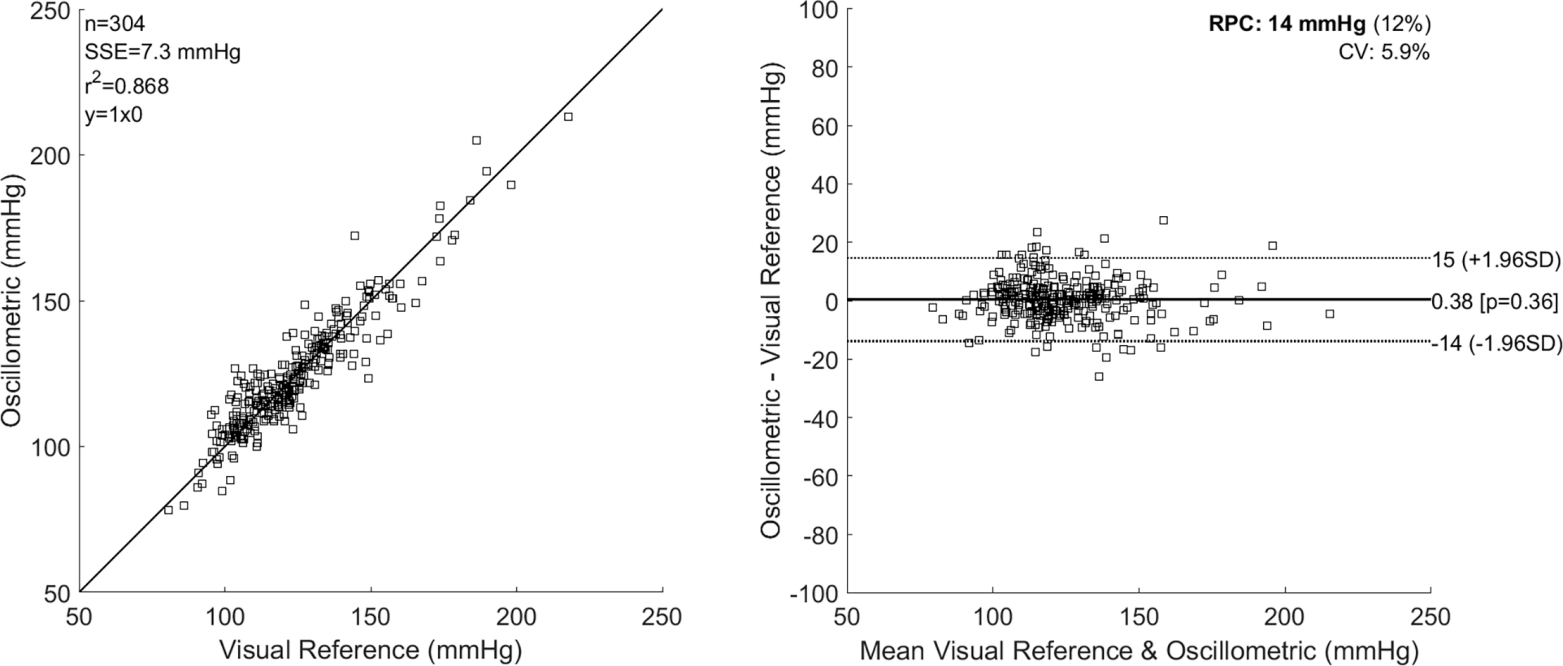
Bland-Altman plots comparing oscillometric estimates of systolic pressure using the characteristic ratio of Rs = 0.565, against reference auscultatory systolic pressure.

In Fig 4, we plot the correlation between the oscillometric systolic pressure for Rs = 0.565, and the reference systolic pressure determined using the automated algorithm as shown in Fig 3. We also calculate the SSE, the Pearson r-value squared (r^2^), the Spearman rho value (ρ), the reproducibility coefficient (1.96*SD) (RPC), the coefficient of variation (CV) as the standard deviation of mean values in %, and the 95% confidence limits on the differences. Similar Bland-Altman plots were generated for all values of Rs tested.

The results obtained for a range of values of Rs spanning the range of 0.45–0.73 reported in the literature [16] are given in Table 2.

**Table 2:** Bland Altman statistics and BHS classification for a range of values of Rs. The row labelled ALG gives the results obtained using an automated algorithm.

Rs	RPC mmHg (1.96*SD)	Spearman rho (ρ)	SSE mmHg	Mean Diff, mmHg	% Diff ≤ 5 mmHg	% Diff ≤ 10 mmHg	% Diff ≤ 15 mmHg	BHS Class
0.4500	14.5	0.9011	10.2	6.9	38.8	71.4	86.2	D
0.4624	13.9	0.9130	9.4	6.2	42.8	74.3	87.2	C
0.5000	13.7	0.9143	8.1	4.1	53.0	81.6	91.8	B
0.5500	14.0	0.9077	7.3	1.2	58.9	86.2	93.1	B
0.5600	14.2	0.9043	7.3	0.7	61.5	85.5	93.1	B
0.5650	14.3	0.9031	7.3	0.4	61.8	85.2	93.4	B
0.5700	14.4	0.9029	7.3	0.1	60.2	85.2	93.1	B
0.5750	14.4	0.9023	7.1	-0.2	59.9	84.9	93.1	B
0.6000	15.1	0.8928	7.9	-1.8	54.9	81.3	91.8	B
0.6491	16.0	0.8802	7.9	-4.8	42.8	70.7	88.8	C
0.7300	17.5	0.8519	13.7	-10.0	20.1	41.4	69.4	D
ALG	10.2	0.9748	5.2	-0.5	83.2	96.1	98.0	A

From Table 2 little difference is shown in RPC, Spearman rho, SSE of mean difference for values 0.56 ≤ Rs ≤ 0.57, however the % Diff ≤ 5 mmHg is a maximum for a value of Rs = 0.565.

## Discussion and conclusion

We reported in the introduction that the MAA is most commonly used in oscillometric NIBP monitors, although many other BP estimation algorithms have been proposed [20–23, 26–29]. However the best values for the characteristic ratios used in the MAA algorithm are disputed, with values for Rs and Rd reported in the literature [16] as between 0.45 and 0.73 and 0.69 to 0.83, respectively. Moreover, characteristic ratios and curve fitting algorithms used in commercially available NIBP monitors are trade secrets and are never reported, a situation that may need to be reviewed by the regulatory and standards bodies.

We have developed and report on a method that allows the accurate determination of characteristic ratios using a Fluke industry standard NIBP analyser, which provides reproducible dynamic blood pressure simulations, designed to verify the performance claims of different NIBP monitors. Using this method, we concluded that the Fluke analyser implements the MAA algorithm with characteristic ratios fixed at Rs = 0.4874±0.0178 and Rd = 0.6577±0.0626, and that the three NIBP monitors manufactured in China implemented the identical MAA algorithm and characteristic ratios. The three NIBP monitors manufactured in Germany and Japan however implemented a different algorithm with significantly larger values of Rs and characteristic ratios that were incompatible with the Fluke analyser. As a result, as we have demonstrated in Table 1, of six oscillometric NIBP tested only three provided outputs consistent with the set simulator values. Clearly, the remaining three devices either did not implement the MAA algorithm or used significantly different characteristic ratios.

Since the characteristic ratio Rs used in the Chinese manufactured NIBP monitors were significantly less than the optimal value of 0.565 and the characteristic ratio Rs for the Japanese manufactured NIBP monitors were significantly greater, we can conclude that the former would overestimate and the latter underestimate the true systolic pressure.

We have demonstrated, as shown in Fig 3, that digital sampling and applying simple signal processing to the auscultatory and oscillometric signal allows the unequivocal determination of systolic pressure, as the first Korotkoff sound that exceeds a background noise threshold. This is entirely analogous but more accurate than, the recommended method based on listening for the first Korotkoff sound.

Since we also record the oscillometric wave, it is then relatively straightforward to use the MAA algorithm to determine the equivalent oscillometric systolic pressure for any chosen characteristic ratio Rs. The record thus obtained as shown in Fig 3, completely characterises the NIBP record and provides additional information such as background noise levels, and the existence of signal artefact and cardiac arrhythmia that would otherwise not be available using a stethoscope. We note that in Fig 3 the SBP calculated using the oscillometric MAA algorithm was 111 mmHg, whilst the SBP determined at the first Korotkoff sound was 103 mmHg, a difference of 8 mmHg.

Using a database of 304 recordings from 102 patients, we demonstrated that the characteristic ratios used for all devices tested, whilst border line acceptable (Grade C) using the BHS grading system, was not optimal, with the best results (Grade B) obtained for a value of 0.5 ≤ Rs ≤ 0.6. Interestingly, Geddes, an early pioneer [14] reported that Rs decreased from 0.57 to 0.45 over an auscultatory systolic pressure ranging from 100 to 190 mm Hg, and was 0.55 at 120 mm Hg. Relative to the optimal value of Rs = 0.565, the Chinese manufactured NIBP devices overestimate the systolic pressure by 6.2 mmHg and the remaining three NIBP devices underestimate the systolic pressure by 4.8mm.

However, the best results presented in this study were obtained from an automated algorithm developed in our laboratory which resulted in a mean difference of -0.5 mmHg, SSE of 5.2 mmHg, r^2^ and Spearman ρ values exceeding 0.93 with 83.2% of results in error by less than 5 mmHg. This algorithm was developed to demonstrate that with relatively simple signal processing, a far more accurate estimate of systolic pressure can be obtained than from the oscillometric MAA method. Unlike the oscillometric method which is based on an OMWE of untested physiological meaning, the modified auscultatory method is based on solid physiological grounds, the first appearance of the Korotkoff sound as the cuff deflates.

Additional advantages of our modified auscultatory method are that algorithms can be readily implemented for the identification of measurement noise or signal artefact [30] and cardiac arrhythmia [31]. Once these are identified, the measurement can be either aborted or the impact of the artefact minimized using signal processing methods. In either case, the systolic pressure estimate can be reported with greater confidence. In the oscillometric method, any measurement noise, signal artefact or cardiac arrhythmia can significantly distort the OMWE, with limited opportunity for redress.

Clearly, the data panel shown in Fig 3 provides ample opportunity for developing algorithms for the robust estimation of diastolic pressure. However, there is poor agreement on what constitutes the diastolic pressure point, and the standard definition, as either cessation or muffling of the Korotkoff sounds in the context of the data panel available in Fig 3, does not provide a sufficiently robust basis on which to develop and test numerical algorithms. Additional research simultaneously comparing data available from NIBP recordings with intra-arterial measurements will be required to resolve this important issue.

Numerous standards including those recommended by the American Thoracic Society (ATS) and the AAMI are based on performance requirements for which the most reasonable test signals are those that represent actual patients. As an example, to test and qualify spirometers for compliance with the ATS standard, a set of 24 standard waveforms have been selected from a larger set of forced vital capacity (FVC) manoeuvres performed by patients who have a diversity of abnormalities and demonstrate a range of test results. Common spirometric parameters are known for each waveform; therefore, the adherence to performance requirements can be readily verified.

Such an approach is long overdue in the testing of NIBP monitors. The availability of a number of digital databases of NIBP recordings with and without abnormalities such as measurement noise, signal artefact or cardiac arrhythmia could provide a reference against which all manufacturers would need to test and report the performance of their devices against an internationally agreed standard. At present, it is clear that whilst all NIBP monitors on the market are meant to comply with ISO 81060–2:2013(E), the AAMI equivalent [9] or the BHS standard [10], little is known publically of the testing regimes used by manufacturers to comply with these standards, which from the clinical perspective may in many cases be inadequate as demonstrated in this study.
